# Comparative Analysis of Coding and Non-Coding Features within Insect Tolerance Loci in Wheat with Their Homologs in Cereal Genomes

**DOI:** 10.3390/ijms222212349

**Published:** 2021-11-16

**Authors:** Tugdem Muslu, Bala Ani Akpinar, Sezgi Biyiklioglu-Kaya, Meral Yuce, Hikmet Budak

**Affiliations:** 1 Molecular Biology, Genetics and Bioengineering Program, Faculty of Engineering and Natural Sciences, Sabanci University, Istanbul 34956, Turkey; tugdem@sabanciuniv.edu (T.M.); sbiyiklioglu@sabanciuniv.edu (S.B.-K.); 2 Montana BioAgriculture, Inc., Missoula, MT 59802, USA; aniakpinar@gmail.com; 3 Sabanci University Nanotechnology Research and Application Centre (SUNUM), Istanbul 34956, Turkey; meralyuce@sabanciuniv.edu

**Keywords:** *WSS*, *OWBM*, resistance, genome organization, wheat, cereals, miRNA

## Abstract

Food insecurity and malnutrition have reached critical levels with increased human population, climate fluctuations, water shortage; therefore, higher-yielding crops are in the spotlight of numerous studies. Abiotic factors affect the yield of staple food crops; among all, wheat stem sawfly (*Cephus cinctus* Norton) and orange wheat blossom midge (*Sitodiplosis mosellana*) are two of the most economically and agronomically harmful insect pests which cause yield loss in cereals, especially in wheat in North America. There is no effective strategy for suppressing this pest damage yet, and only the plants with intrinsic tolerance mechanisms such as solid stem phenotypes for *WSS* and antixenosis and/or antibiosis mechanisms for *OWBM* can limit damage. A major QTL and a causal gene for *WSS* resistance were previously identified in wheat, and 3 major QTLs and a causal gene for *OWBM* resistance. Here, we present a comparative analysis of coding and non-coding features of these loci of wheat across important cereal crops, barley, rye, oat, and rice. This research paves the way for our cloning and editing of additional *WSS* and *OWBM* tolerance gene(s), proteins, and metabolites.

## 1. Introduction

Ensuring global food security and agricultural sustainability becomes increasingly challenging every year, with the pressure of a continuously growing population causing a rise in food demand, dietary changes and shortage of land, and water resources [1,2]. Global food demand is estimated to increase by 70–85% by 2050 [3]; therefore, obtaining higher-yielding crops is vital to meet the food demand by producing more products with fewer resources. Wheat is the most consumed cultivated crop globally, contributing 20% of the calories consumed by humans [4]. The United Nations’ Food and Agriculture Organization (FAO) estimated the global wheat production to be around 776 million tons in the 2021/2022 marketing season and holds a USD 50 billion trade market. The United States of America annually exports about 28 million metric tons of wheat, majorly produced in Montana, Kansas, and North Dakota. It is predicted that the wheat demand will increase by 60% by 2050 as the world population rapidly increases [5,6].

While the food demand is continuously increasing, there are also a variety of stress factors threatening agricultural productivity. Abiotic stress conditions such as drought, high salinity, high or low temperatures, and heavy metals can negatively affect crop productivity and are often interrelated [7]. Additionally, plants are vulnerable to diseases caused by biotic stress factors such as viruses, fungi, bacteria, weeds, nematodes, arachnids, and insects [8]. Two such pests, wheat stem sawfly (*Cephus cinctus* Norton, *WSS*) and orange wheat blossom midge (*Sitodiplosis mosellana, OWBM*) can cause significant economic losses through yield deprivation and damaged kernels that cannot be harvested in cereals.

*WSS* is an endemic stem-mining insect of Northern Great plains and a significant pest of winter and spring hexaploid wheat and tetraploid durum wheat, minimizing its yield and causing economic losses [9,10]. Severe sawfly infestations occur especially in Montana, North Dakota, northern South Dakota, and western Minnesota, and there are currently no chemical control measures to suppress *WSS* damage [11].

The *WSS* produces one generation per year when the female sawflies oviposit an average of 30–50 eggs into the internode of the host plant stem, and only one larva survives [10]. Larvae feed on parenchyma and vascular bundles and move down the stem as they mature. This larval feeding decreases the photosynthetic ability of the host plant and lowers the mass by up to 30% [12]. Eventually, larvae cut the stem at the base creating a lodge to allow them to accumulate during overwintering diapause [13]. Infested stems are easily windblown and not easily picked during harvest, resulting in further yield loss [12]. In addition, uncut infested plants suffer yield loss due to a decrease in head weight [9]. Plant characteristics influence the host preference of female sawflies, which is important in progeny survival as the *WSS* larva cannot switch hosts [10]. Although sawfly may oviposit in other cereals, including oat, rye, and barley, larvae development rarely occurs in barley and rye, whereas larvae do not survive in oat [14]. Host–plant resistance provided by solid-stemmed and semi-solid-stemmed cultivars is the only effective management strategy against *WSS* infestation as pith development in the culm lumen reduces larva survival and interferes with insect oviposition [15]. A major QTL, *SSt1,* identified on the long arm of the 3B chromosome has been associated with stem solidness in tetraploid durum wheat, common wheat, and wild emmer wheat [12]. This QTL and its orthologue in hexaploid wheat, *Qss.msub-3BL,* have been the most favorable tools of management against *WSS* in wheat [16]. Recently, the *TdDof* gene (TRITD3Bv1G280530) has been identified as the causal gene of the stem solidness phenotype conferred by the *SSt1* QTL in tetraploid wheat. Copy number variation, where the presence of additional copies coupled with increased expression of this gene, has been shown to positively regulate stem solidness [17].

Orange wheat blossom midge (*OWBM*) is another damaging pest of spring wheat effective in a wide range from North America, to several European countries and to Asia [18]. Around May, the mature *OWBM* larvae drop from the wheat ears to the ground, resulting in diapause of the larvae within a cocoon for a long time. In springtime, the larvae leave the cocoon and pupate at the soil surface. Two weeks after they pupate, adult midges move to the wheat canopy, where female midges lay up to 80 eggs. The hatched larvae will eat the kernels damaging the crop [19,20]. There are two host resistance mechanisms against *OWBM* damage: (1) abnormal or prevented oviposition (antixenosis) and (2) suppression of larval growth (antibiosis) [19]. *Sm1* gene mapped on the short arm of chromosome 2B from Augusta cultivar of American wheat has been the first antibiosis gene identified to provide resistance against *OWBM*. *Sm1* is believed to inhibit larval growth through ferulic-acid and/or p-coumaric acid production in seed coat [21,22]. The QTL containing the *Sm1* gene has been under close scrutiny and had been saturated by several molecular markers by Kassa et al. (2016) [19]. Finally, very recently, extensive genome sequencing in wheat led to the identification of a candidate gene for this locus, an NB-ARC-LRR-kinase-MSP gene, that is likely responsible for the *OWBM* resistance phenotype [23].

Even though the antibiosis-related *Sm1* gene has been the only resource utilized in *OWBM* management strategies in the field so far, antixenosis-related mechanisms have been observed in common and durum wheat that can also be utilized to grow resilient crops [24,25]. Accordingly, a major QTL on chromosome 1A, *QSm.mst-1A*, was first identified in 2011 in the spring wheat variety Reeder [26]. This QTL was associated with oviposition deterrence again in a recent study and was saturated with molecular markers to facilitate cloning and marker-assisted selection efforts. Finally, another QTL on chromosome 4A has attracted attention for potential use against *OWBM* through oviposition deterrence [25]. So far, the causal genes underlying the antixenosis responses conferred by these QTL are unknown.

In this study, we compared the content and organization of coding sequences within the QTL identified on 3B, 2B, 1A, and 4A chromosomes that confer resistance against *WSS* or *OWBM* across cereals wheat, barley, rye, and rice. In addition, we identified putative microRNA (miRNA) encoding sequences within these QTLs that may contribute to the resistance response. Rather than focusing on causal or candidate genes, we sought to explore how these chromosomal regions carrying important information on insect tolerance were shaped in closely related cereal genomes. We also explored sequences within the newly released oat genome assembly that should belong to the genomic loci homologous to these insect tolerance loci.

## 2. Results

### 2.1. The Content and Organization of the Coding Features around the SSt1 Locus Appear to Be Widely Conserved across Homologous Regions in Cereal Genomes

The causal gene, *TdDof*, residing within the major QTL on the long arm of chromosome 3B (*Qss.msub-3BL* in hexaploid bread and *SSt1* in tetraploid wheat; 3BL-QTL hereafter, for simplicity) that confers stem solidness in wheat has been recently identified [17]. In order to understand how this locus has been broadly shaped in cereal genomes, molecular markers defining the 3BL-QTL [12] were first mapped to the 3B chromosomes in *Triticum aestivum* cv. Chinese spring (hexaploid, AABBDD, Chinese Spring hereafter) and *Triticum turgidum* ssp. *durum* cv. Svevo and *Triticum turgidum* ssp. *dicoccoides* genotype Zavitan (tetraploid, AABB, Svevo, and Zavitan, respectively, hereafter) and to the homologous 3H chromosome in *Hordeum vulgare* cv. Morex (barley). Additionally, due to known rearrangements in the rye genome, the molecular markers were mapped to the entire genome of *Secale cereale* line Lo7 (rye) [27]. Similarly, due to the evolutionary distance and the small genome size, molecular markers were mapped to the entire *Oryza sativa* ssp. *japonica* cv. Nipponbare genome (rice) [28]. Moreover, 3BL-QTL molecular markers were compared against the recently released genome assembly of *Avena sativa* (oat) [29]. Using these markers, homologous regions on wheat 3B chromosomes, barley 3H chromosome and rye 6R chromosome were identified, spanning 12–27 Megabases (Mb) (Supplementary Table S1). Due to increased evolutionary distance in rice, a potential locus homologous to the 3B-QTL could not be identified through the molecular markers.

**Figure 1:**
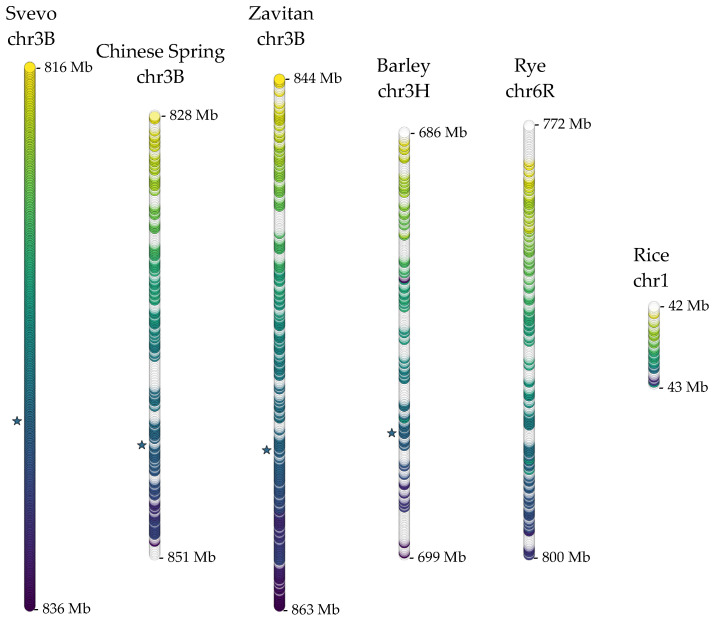
The coding organization along the 3BL-QTL in Svevo chromosome 3B and homologous regions on Zavitan 3B, Chinese Spring 3B, barley 3H, rye 6R, and rice 1 chromosomes. Stacked circles indicate genes ordered along each chromosomal segment, and colors only indicate homologous relationships to Svevo genes; genes without Svevo homologs are not colored. Color patterning is for visual purposes. The lengths of the chromosome segments are not proportionate to their physical lengths but rather to the total number of genes within each segment. Stars indicate the causal gene (TRITD_3Bv1G280530) in the Svevo 3BL-QTL and its homologs in the other chromosomal segments, if present.

Chromosome subsequences for the given intervals were extracted for a comparative analysis of coding and non-coding features. For coding features, each gene’s longest isoform within the subsequences was retrieved from respective GFF files. With this approach, 309 transcripts for Chinese Spring, 316 transcripts for Svevo, 258 transcripts for Zavitan, 248 transcripts for barley, and 252 transcripts for rye were extracted. These transcripts were first used to identify the homologous region in the rice genome, which could not have been determined using the 3BL-QTL molecular markers. For this, all the transcript isoforms were combined and blasted against all rice coding sequences. Of the significant hits, transcripts between Os01t0958700-01 and Os01t0977200-00 on rice chromosome 1, spanning ~1Mb, were determined to be homologous to the 3BL-QTL in wheat. This interval contains 46 rice genes. Potential functions of the transcripts from all cereal loci were deduced based on homology to annotated proteins from the Uniprot database and/or fully annotated proteome of *Brachypodium distachyon*, the model plant for monocots (Supplementary Table S2). Furthermore, the protein sequences identified in the homologous regions were compared to unpublished proteomics data of semi-solid/solid-stemmed wheat cultivars upon infestation (Supplementary Table S3). Since the causal gene for stem solidness was first described in the Svevo genome, we compared the organization of these coding features within each homologous chromosomal interval in the cereal genomes with respect to Svevo, which indicated a high level of overall conservation across all regions. The distal regions of each homologous interval seemed to be more prone to the rearrangements, where conservation also tended to be lost as the evolutionary distance increased (Figure 1).

Currently, only a fragmented genome assembly is available in oat, which restricts direct comparisons of the genomic organization of homologous loci or features. However, molecular markers defining the 3BL-QTL matched 12 contigs that may represent a homologous region in the oat genome. Additionally, the transcript sequences identified from homologous regions on wheat, barley, rye, and rice chromosomes matched 172 oat contigs. In total, 176 oat contigs may be associated with the genomic loci that are homologous to wheat *SSt1* (Supplementary Table S4).

### 2.2. Several Precursor Sequences Are Predicted from the SSt1 and Homologous Loci in Cereals That Encodes miRNAs, Which Are Widely Conserved at the Family Level

**Figure 2:**
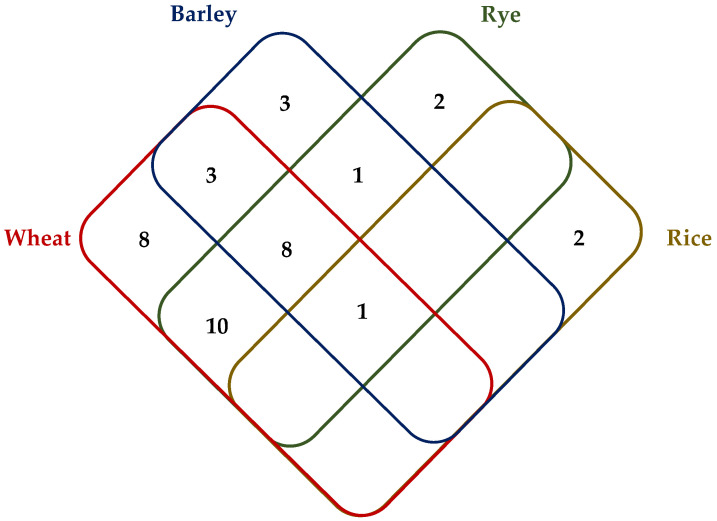
Thirty-eight miRNA families identified from homolog QTL of *WSS* 3B in Chinese Spring, barley, rye, rice, Svevo, and Zavitan. Chinese Spring, Svevo, and Zavitan miRNA families are grouped and shown as wheat miRNA families. The numbers indicate the number of miRNA families identified in each cereal species that are common (or not) with the others.

Homology-based miRNA identification from the given intervals of the chromosome subsequences indicated a total of 485 precursor miRNA sequences for 38 miRNA families. miR1122, miR1137, miR1120, miR1127, and miR5049 families were the miRNA families with the most abundant precursor miRNA sequences (Supplementary Table S5). The number of miRNA families identified in Chinese Spring, Svevo, Zavitan, and rye was found to be close to each other, 23, 26, 23, 22, respectively, of which 16 were common to all those species. Relatively fewer, 16 miRNA families were identified from barley chromosome subsequence, and in rice, there were three identified miRNA families. miRNA families identified from homologous 3BL QTL were mostly conserved between closely related wheat, barley, and rye. While the relatively smaller region that could be identified in rice did not permit meaningful comparisons with other cereals, of the three families that could be identified in rice, miR1130 was common to all. We have identified eight miRNA families present only in wheat genotypes (Figure 2). All miRNA families identified in 3BL QTL regions have been shown in Supplementary Table S6. mRNA target analysis of the putative miRNAs has shown that miR1127 and miR1439 of Chinese Spring, miR1118 of Svevo, and miR5049 of Zavitan have potential targets within the same 3BL-QTL homologous regions of each respective genotype. The transcript targets were then compared to all *Viridiplantae* proteins to reveal any annotated homologs. miR1118 from Svevo is predicted to target a Pik2-like protein which may be involved in insect tolerance while the other miRNA targets are mostly uncharacterized proteins (Supplementary Table S7).

### 2.3. Both the Content and Organization of the Coding Features across Genomic Regions Homologous to Sm1 Locus Suggest Considerable Rearrangements between Rye and Other Triticeae Genomes

**Figure 3:**
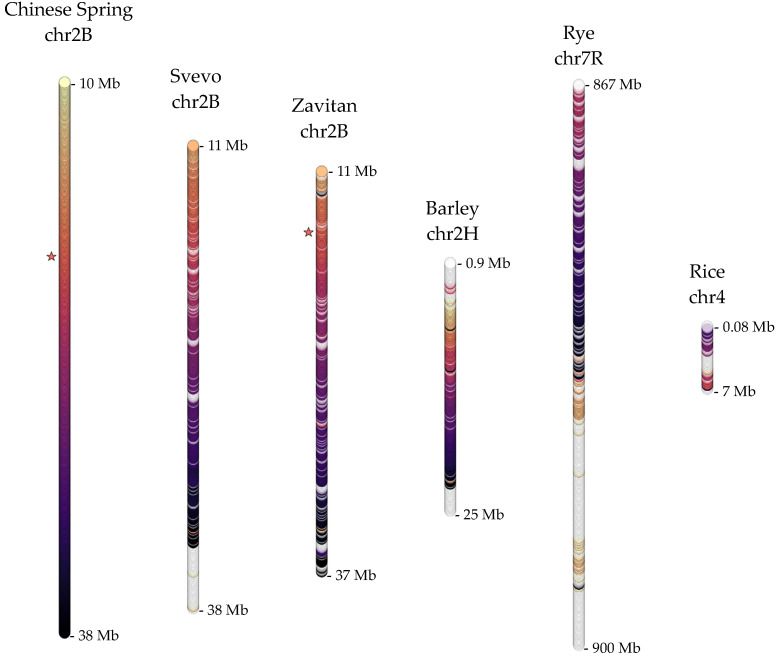
The organization of the coding features along the *Sm1*-homologous regions in Chinese Spring chromosome 2B, Svevo chromosome 2B, Zavitan chromosome 2B, barley chromosome 2H, rye chromosome 7R, and rice chromosome 4. Colors and patterning are as in Figure 1, except in reference to Chinese Spring. Stars indicate the best Chinese Spring match (TraesCS2B03G0071700.2) to the *Sm1* candidate gene described in CDC Landmark in Walkowiak et al. [23] and its homolog in Zavitan 2B.

Extensive genome sequencing in diverse wheat genotypes has led to identifying the candidate gene for the *Sm1* locus on chromosome 2B, conferring resistance to another devastating pest, *OWBM* [23]. In order to explore the genomic organization of this locus on cereal genomes, molecular markers associated with *Sm1* were mapped on Chinese Spring 2B, Svevo 2B, Zavitan 2B, barley 2H chromosomes, as well as rye and rice genomes. While homologous regions of 25–33 Mb in length were identified in wheat 2B and rye 7R chromosomes, too few molecular markers were mapped to the barley 2H chromosome and the rice genome (Supplementary Table S1). For barley and rice, transcript isoforms from wheat (452, 380, and 331 isoforms from Chinese Spring, Svevo, and Zavitan 2B chromosomes, respectively) and rye (460 isoforms from 7R chromosome) homologous regions were used to identify homologous regions of 24.9 Mb on barley 2H chromosome and 7.2 Mb on rice chromosome 4, spanning 204 and 52 genes, respectively (Supplementary Table S1). Potential functions of these transcripts were inferred based on homology to annotated proteins from the Uniprot database, fully annotated proteome of *B. distachyon*, and an unpublished proteomics study of semi-solid and solid stemmed wheat cultivars (Supplementary Tables S3 and S8). The organization of the genes in the homologous regions was compared with respect to Chinese Spring since the candidate gene was first defined in the hexaploid background [23]. With respect to Chinese Spring, extensive rearrangements, including an apparent inversion was evident in the rye 7R chromosome. The conservation of the coding features in the proximal regions of *Sm1* also appeared to be lost in rye 7R (Figure 3).

The comparison of the oat genome assembly to the molecular markers associated with the *Sm1* locus identified three contigs. These contigs were among the 276 oat contigs that also significantly matched transcript sequences from wheat, barley, rye, and rice chromosomal regions belonging to or homologous to the *Sm1* loci. These 276 may represent a genomic locus in the oat genome that is homologous to the *Sm1* locus, even though it is not yet possible to deduce the genomic organization of this region compared to its relatives due to the fragmented nature of the current oat genome assembly (Supplementary Table S4).

### 2.4. Genomic Loci Homologous to the Sm1 Locus in Cereals Putatively Contain Several miRNA Precursors

**Figure 4:**
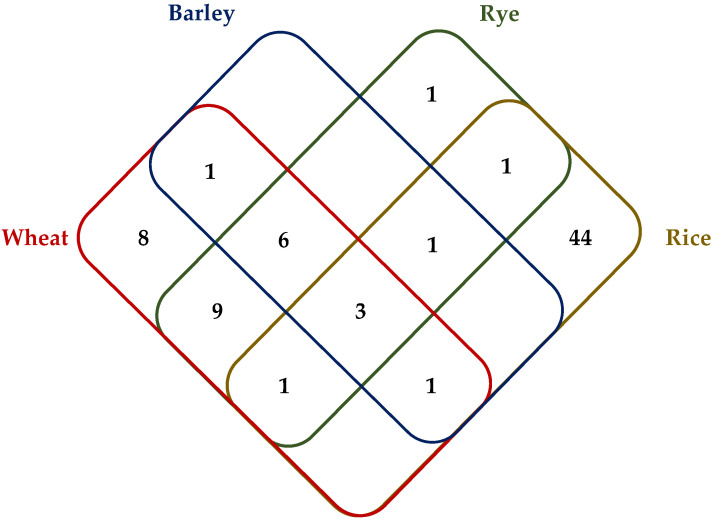
Seventy-six miRNA families identified from Chinese Spring, Svevo, Zavitan (all shown as wheat), barley, rye, and rice homologs of the 2B QTL region. Apart from 44 miRNA families identified only in rice, the remaining 22 miRNA families are primarily conserved in wheat, barley, and rye. The numbers indicate the number of miRNA families identified in each species that are common (or not) with the others.

miRNA identification analysis from Chinese Spring, Svevo, Zavitan, barley, rye, and rice chromosome subsequences yielded 24, 25, 25, 12, 22, and 51 miRNA families, respectively, resulting in a total number of 76 miRNA families with 659 precursor sequences (Supplementary Table S5). miR1122, miR1120, and miR5049 were found to have the highest number of precursor miRNA sequences, and miR1128 and miR1130 were identified in all chromosome subsequences. Chinese Spring, Svevo, and Zavitan genotypes have miR1121, miR1125, and miR1136 families not identified in barley, rye, and rice, whereas miR1439 was only identified in barley, rye, and rice (Supplementary Table S6). Figure 4 shows the conservation of miRNA families among the species analyzed. Three miRNA families were commonly predicted from all cereal loci analyzed. There is a vast amount of miRNA families, 44 miRNA families, predicted only in rice. We identified eight miRNA families only present in wheat genotypes. psRNA target analysis of mature miRNA sequences of putative miRNA families against coding sequences of homologous regions revealed five miRNA families—miR1120, miR1127, miR1135, miR2118, and miR9782—with at least one transcript target mapped on these homologous regions. Putative targets of miR2118 in Zavitan were predicted to be a disease resistance protein RPM-like gene, whereas a *Morf Related Gene 1* (MRG-1)-like protein was predicted to be targeted by miR1127 of Chinese Spring and Zavitan. Functional aspects of the putative targets of other 3 miRNA families remain mostly unidentified as the target transcripts have not shown homology to well-characterized plant proteins (Supplementary Table S7).

### 2.5. A Comparison of the Coding Features across Genomic Regions Homologous to Additional Loci Associated with OWBM Resistance Suggests Evident Inversions in One or More Cereal Genomes

**Figure 5:**
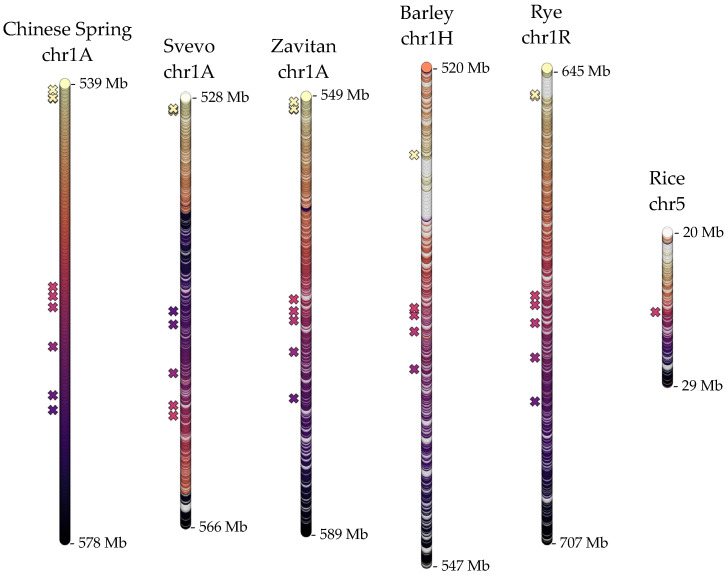
The organization of the coding features along an additional QTL for oviposition deterrence against *OWBM*, on wheat 1A chromosomes and homologous regions on barley chromosome 1H, rye chromosome 1R, and rice chromosome 5. Colors and patterning are as in Figure 3, in reference to Chinese Spring. ‘X’ symbols indicate genes homologous to potential candidates for the 1A-QTL proposed in a recent study [18].

In addition to the antibiotic resistance provided by the *Sm1* locus, additional loci have also been associated with *OWBM* resistance through different mechanisms. One known major QTL, associated with oviposition deterrence, resides on chromosome 1A in wheat [18,26]. Using molecular markers mapped on this 1A-QTL [18], regions of 28–62 Mb homologous to this QTL were identified in wheat chromosomes 1A, barley chromosome 1H, and rye chromosome 1R (Supplementary Table S1). In rice, homologous transcripts from wheat (582, 545, and 556 isoforms on Chinese Spring, Svevo, and Zavitan 1A chromosomes, respectively), barley (633 isoforms on 1H), and rye (602 isoforms on 1R) identified a 9 Mb region on chromosome 5, that contained 193 genes. The organization of these genes along the homologous regions, with respect to Chinese Spring 1A, suggested overall conservation of the gene sequences and orders, except for an apparent inversion in Svevo 1A (Figure 5).

**Figure 6:**
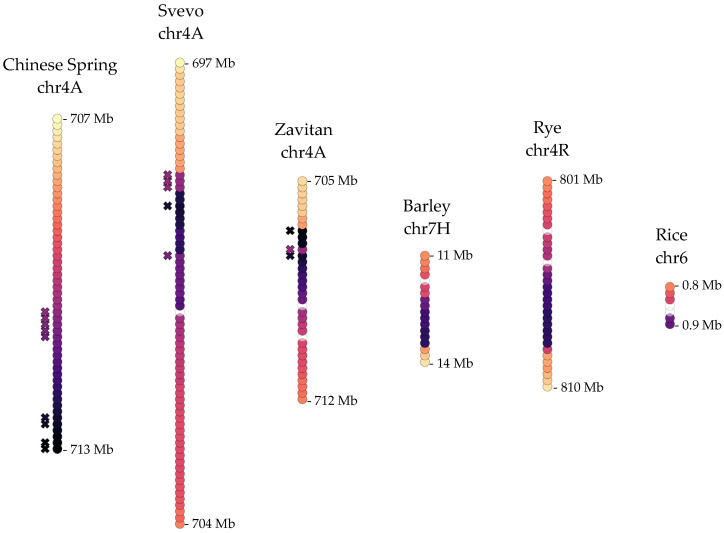
The organization of the coding features along a second QTL for oviposition deterrence against *OWBM*, on wheat 4A chromosomes and homologous regions on barley chromosome 7H, rye chromosome 4R, and rice chromosome 6. Colors and patterning are as in Figure 3, in reference to Chinese Spring. ‘X’ symbols indicate genes homologous to potential candidates for the 4A-QTL proposed in two recent studies [25,30].

Another major QTL for *OWBM* resistance was mapped to the long arm of chromosome 4A in wheat, where the candidate gene has not been identified yet [25,30]. While relatively few markers have been mapped to this resistance loci, a 4.9 Mb genomic region has been defined on the previous assembly of the Chinese Spring genome (IWGSC v1.0 assembly) that included 58 genes [25]. Therefore, the longest isoforms encoded by these genes were used to identify the transcripts that may be associated with the 4AL-QTL in the current version of the Chinese Spring genome (IWGSC v2.1 assembly) as well as homologous transcripts in Svevo, Zavitan, barley, rye, and rice. These transcripts identified 5.49–9.5 Mb regions on homologous wheat 4A and rye 7R chromosomes and a relatively small region of only 3.75 Mb on the barley 7H chromosome. In rice, only a few homologous transcripts were identified on chromosome 6 (Supplementary Table S1). These regions contained 54 genes in Chinese Spring 4A, 75 genes in Svevo 4A, 36 genes in Zavitan 4A, 18 genes in barley 7H, 34 genes in rye 4R, and 7 genes in rice chromosome 6. Comparative analysis indicated shared patterns of rearrangements in durum wheat Svevo and Zavitan and rye and barley (Figure 6). Putative functions for the transcripts identified in 1A-QTL and 4AL-QTL homologous loci are given in Supplementary Tables S3 and S8.

The comparison of molecular markers associated with the 1A-QTL to the oat genome assembly identified 18 contigs that may belong to a homologous region on the oat genome. Curiously, homologous transcripts identified in wheat, barley, rye, and rice matched a baffling total of 4659 contigs from the oat genome assembly. For the 4A-QTL, where relatively small genomic regions were comparatively analyzed, transcripts combined from all homologous loci identified 12 contigs that may represent a homologous region in the oat genome (Supplementary Table S4).

### 2.6. The Comparison of miRNAs across Genomic Regions Homologous to Additional Loci Associated with OWBM Resistance in Cereal Genomes

**Figure 7:**
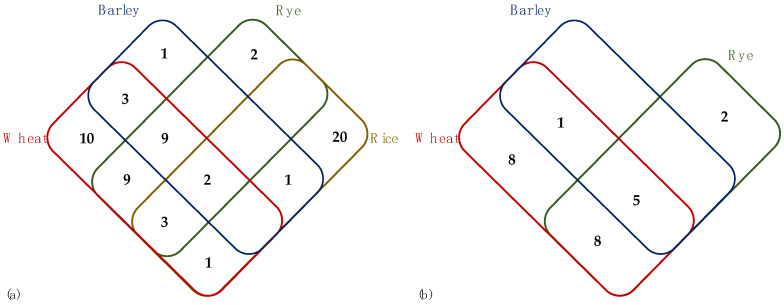
(**a**) Distribution of 61 miRNA families identified in 1A-QTL homologs among wheat genotypes, barley, rye, and rice. Each number indicates the number of miRNA families identified in each cereal species that are shared (or not) with the others. Three miRNA families were shown to be shared among all analyzed species, whereas eight miRNAs were conserved in wheat, barley, and rye. (**b**) Distribution of 24 miRNA families identified in 4A-QTL homologs among wheat genotypes, barley, and rye. The number of miRNA families identified in each cereal and the number of miRNA families common to each other are indicated by each number. Due to smaller chromosome subsequences, 4A-QTL yielded relatively fewer miRNA families compared to other QTLs. Among 24 miRNA families, wheat and rye share 13 miRNA families, whereas barley has 6 common miRNA families with wheat.

Sixty-one miRNA families with 746 precursor miRNA sequences were identified from chromosome subsequences of 1A-QTL homologous regions, consisting of 35 miRNA families identified from Chinese Spring, 35 and 32 miRNA families from Svevo and Zavitan, respectively. A relatively smaller number of miRNA families, 27 for rice, 25 for rye, and 16 miRNA families for barley chromosome subsequences, were identified. miR1120, miR1122, miR5049, miR1127, and miR1137 are the miRNA families with the highest number of pre-miRNA sequences (Supplementary Table S5). Similar to the results of homologous 2B-QTL miRNA identification, many rice miRNA families were identified. Ten miRNA families were identified to be present only in wheat genotypes. Rye has been found to have more common miRNA families with wheat genotypes than barley (Figure 7a). A comparison of all mature miRNA sequences against all transcripts identified from the respective homologous loci suggested that, overall, nine miRNA families may target transcripts from the same loci. Even though the majority of these transcripts did not have functionally well-characterized homologs among *Viridiplantae*, putative targets of miR1118, miR1439, and miR2275 had matches to genes that may be involved in disease resistance. Specifically, putative miR2275 targets were highly similar to resistance gene analog (RGA) type of genes, whereas the miR1118 and miR1439 families putatively targeted PIK-like isoforms. It should be noted that putative miR1439 targets in barley solely matched uncharacterized proteins, which indicate that these may include resistance-associated genes that are yet to be characterized (Supplementary Table S7).

Due to the low number of 4AL-QTL homologous transcripts identified in rice, miRNA identification was performed by excluding rice chromosome subsequences. A total of 224 precursor miRNA sequences resulted in total of 24 miRNA families, which composed of 17 miRNA families identified from Chinese spring, 18 from Svevo, 17 from Zavitan, 6 miRNA families from barley and 15 from rye chromosome subsequences, and 5 of those miRNA families, miR1127, miR1122, miR5049, miR1120 and miR1130 are found to be common to all species (Supplementary Tables S5 and S6). Figure 7b shows how the identified miRNA families are shared among wheat genotypes, barley, and rye. Eight miRNA families were identified only in wheat genotypes, and the remaining miRNAs are mostly conserved among all analyzed species. None of the predicted miRNA families targeted transcripts on the 4A-QTL homologous regions.

## 3. Discussion

Food security of the upcoming generations depends on our ability to develop resilient crops not only to the more frequent and severe weather extremes caused by global warming but also to the devastating biotic threats by pests and pathogens that are constantly under an arms race with their hosts. In this study, we focused on two crucial wheat pathogens, Wheat Stem Sawfly (*WSS*; *Cephus cinctus Norton*) and Orange Wheat Blossom Midge (*OWBM*, *Sitodiplosis mosellana*), and explored the content and organization of coding and non-coding features on multiple resistance loci across cereal genomes. The focus of this study is not the causal or candidate genes, which have recently been firmly established for two of the loci already [17,23], but rather to provide a comparative overview of these loci across essential cereal crops and search cues into how evolution has acted on them.

Modern grass genomes, including cereals, are believed to have evolved from an ancestor with 7 predicted proto-chromosomes, through whole-genome duplications and chromosomal rearrangements [31,32]. The extant species still retain considerable synteny across large chromosomal segments [31,33], to an extent where the syntenic relationships help build reference assemblies and/or inferred gene orders for closely related complex genomes [34]. Functionally important loci can be expected to remain in such well-conserved syntenic loci. Stem solidness trait has been utilized as the primary defense strategy against *WSS* in wheat. The causal gene, *TdDof*, of the major QTL on chromosome 3BL controlling stem solidness was recently identified and characterized [17]. When this QTL and homologous regions in related *Poaceae* species were compared, a high level of synteny was observed (Figure 1). While the sequence conservation of the genes seemed to be lost towards the distal end, the overall order of the genes seemed to be well preserved. The *TdDof* gene is identified in the tetraploid durum wheat and is present in the hexaploid bread wheat genome. A putative homolog was also identified in the barley genome; however, this does not inform upon stem solidness in barley as the copy number, not the mere presence, of *TdDof* regulates pith filling of the stems [17]. In rye, the region homologous to the wheat 3BL-QTL was clearly involved in the ancestral translocation between chromosomes 3 and 6 [27] yet, this ancestral translocation did not seem to disrupt the homology to a great extent (Figure 1). The long arm of chromosome 6R also appears to be enriched in nucleotide-binding site leucine-rich repeat (NLR) genes involved in pathogen resistance [35]. It is, thus, tempting to wonder that, beyond stem solidness, whether this locus has been under purifying selection during the *Poaceae* evolution for its importance in biotic stress responses.

Several genomic loci have also been associated with resistance against another devastating pest, *OWBM*. Most notably, the *Sm1* locus on the short arm of chromosome 2B in wheat has long been known to confer resistance through antibiosis. A candidate gene for this locus has also been recently suggested [23]. Compared to the 3BL-QTL, the *Sm1* locus appeared to be less conserved across *Poaceae* (Figure 3). Despite regions of similar physical length, Svevo and Zavitan might have lost homologous sequences to the proximal and distal parts of the *Sm1* locus in Chinese Spring. Additionally, a major rearrangement might have occurred in the rye, after rye and barley had diverged from their last common ancestor. In rye, complex ancestral rearrangements that translocated the most telomeric part of the short arm of ancestral chromosome 2 to the telomeric part of the long arm of ancestral chromosome 7 might have disrupted the grass homology within this locus. A proposed model that involves multiple steps of translocation to the formation of the modern 7R chromosome may explain the extensive loss of homology for this region [27]. In rice, conservation of only a few genes at the microscale was observed. Interestingly, a homologous sequence to the *Sm1* candidate gene was found only in the wild genotype Zavitan but not in the domesticated Svevo cultivar (Figure 3).

At the sequence level, extensive conservation across cereals was evident for another known QTL on wheat chromosome 1A that provides resistance by interfering with the oviposition of *OWBM* eggs. Nevertheless, an apparent inversion on the Svevo 1A chromosome was observed (Figure 5). A previous study has proposed 11 candidate genes for the 1A-QTL based on functional annotation [18]. Several of these had homologous sequences within each chromosomal segment analyzed, further supporting that these loci may be structurally and functionally very well preserved. The identification of the candidate gene that is responsible for oviposition deterrence will await further experimental studies. Despite limited genomic data on another QTL on wheat chromosome 4A that contributes to *OWBM* resistance [25,30], a comparison of homologous genomic regions in cereals revealed an interesting observation. Genome-specific rearrangements might have occurred twice within *Triticeae*: Once after the hybridization of the D-genome to the tetraploid wheat ancestor, giving rise to the hexaploid bread wheat and once after the divergence of barley and rye from their last common ancestor with wheat (Figure 6). A recent inversion might have shaped the Chinese Spring QTL compared to the tetraploid Svevo and Zavitan, and a more complex rearrangement might have shaped the common ancestor or rye and barley.

The proteomics approach provides a unique chance to investigate plant stress responses in more depth; thus, studies on complex abiotic and biotic stress responses on cereals hold great importance [36]. Putative functional annotation of homologous insect tolerance loci has identified the conserved proteins encoded by the genes in these loci (Supplementary Tables S2, S3, and S8). While the causal genes for 3BL-QTL and 2B-QTL have been identified [17,23] and the 1A-QTL has been under scrutiny for some time [26], the 4A-QTL has very recently been proposed and relatively little is known on this potential QTL that confers oviposition deterrence against *OWBM*. The inferred functions of the proteins encoded by the genes within this QTL may provide interesting insight into potential candidates (Supplementary Table S8). Among these, ENHANCED DISEASE RESISTANCE 2-like was found across all cereal loci, along with growth-related GROWTH-REGULATING FACTOR 5 and SENESCENCE-RELATED GENE 1. In addition, CYCLOPS, usually together with calmodulin-like proteins, was found across all loci. Interestingly, CYCLOPS proteins and calcium and calmodulin-dependent kinases are believed to constitute an ancient signaling complex that is required for infection of symbiotic bacteria [37]. Homologous sequences to the stress-related chaperone ClpB1 [38] were found in wheat and barley. Similarly, sequences homologous to 7-DEOXYLOGANETIN GLUCOSYLTRANSFERASE involved in the biosynthesis of iridoids, important defense compounds against herbivores [39], were found in wheat barley and rye. Of the candidate genes that were proposed in the recent studies [25,30], three (TraesCS4A03G1085400.1, TraesCS4A03G1086100.1, TraesCS4A03G1092700.1; CS annotation v2.1) were similar to BISDEMETHOXYCURCUMIN SYNTHASE, which is involved in flavonoid biosynthesis, had homologs only in wheat genotypes. Another candidate gene, TraesCS4A03G1086200.1, matched 2-HYDROXYISOFLAVANONE DEHYDRATASE. Svevo genes TRITD_4Av1G250060 and TRITD_4Av1G250090 also matched this protein, which is involved in isoflavone biosynthesis and is targeted by bacterial virulence factors to facilitate infection in soybean [40]. The 4A-QTL and homologous loci in cereals may encode genes that are involved in the synthesis of secondary metabolites and defense compounds (Supplementary Table S8).

Non-coding genomes have gained interest after discovering their regulatory functions on the coding parts of the genomes [41]. Many important plant microRNAs have previously been shown to be well conserved among *Viridiplantae* for their vital role as gene regulators. Manipulation of miRNAs by molecular techniques thus holds great promise for the future of economically important crops [42]. Homology-based miRNA identification from the chromosome subsequences of *OWBM* and *WSS* QTLs of Chinese Spring, Svevo, Zavitan, barley, rye, and rice have shown the conservation of miRNA families as well as the emergence of new miRNA families. 1A-QTL and 2B-QTL homologous regions resulted in many rice-specific miRNA families which are not present in other cereals. The small homologous region of 3BL-QTL on rice chromosome 1 resulted in a lower number of miRNA families compared to other loci. Wheat, barley, and rye belong to the *Triticeae* tribe and have a more recent ancestor after divergence with rice [43]. The presence of miRNA families identified only in rice may be explained by their evolutionary distance. In 2A-QTL, 3BL-QTL, and 4A-QTL regions, eight miRNA families, and in 1A-QTL region, ten miRNA families were found to be present in only wheat genotypes. These miRNAs may be emerged after the divergence of wheat from barley and rye.

Only a small fraction of identified miRNAs was found to have a transcript target mapped on the homologous regions of resistance QTLs. Transcript targets of four miRNA families, miR1118, miR1439, miR2118, and miR2275, in some genotypes have been predicted to be disease-resistance gene-like proteins, even though most of these transcripts only matched with uncharacterized proteins (Supplementary Table S7). Plant miRNAs involved in plants’ intracellular resistance mechanisms and miRNA-mediated expression of the insect-related genes are crucial for plant defense against a wide range of pathogens. Identifying such miRNAs will lead to the development of miRNA-based plant resistance mechanisms using molecular techniques and insect-resistant breeding [44]. A more comprehensive analysis of the transcript targets, including those out of QTL regions, may help unravel the function of identified microRNAs in biotic stress conditions [45].

## 4. Conclusions

In this study, we examined the organization of coding and non-coding features of loci associated with *WSS* and *OWBM* resistance in an evolutionary comparative way among cereals. As it is the only known tolerance mechanism for *WSS*, the causal stem solidness gene, *Tdof*, holds great importance. We have compared the homologous regions of the QTL associated with *Tdof* and show a high level of conservation among all species, including the overall order of the genes in the genomic loci. However, evolutionary changes were shown to be more frequent on the distal ends of the homologous regions. Comparative analysis of *Sm1* locus revealed major rearrangements in rye chromosome 7R which resulted in the loss of homology in this region. When compared with respect to Chinese Spring, a homologous sequence to the antibiosis gene, *Sm1*, is only identified in Zavitan genotype, and not in the Svevo cultivar, both have the BBAA genome. We have also described a potential genomic locus in the oat genome which may be homologous to *Sm1*. Homologous region analysis for *1A-QTL*, the locus associated with oviposition deterrence of *OWBM* eggs, presented a potential inversion on the Svevo 1A chromosome even though an overall extensive conservation among cereals was shown. Interestingly, the conservation of *4A-QTL* revealed possible complex rearrangements for *Triticeae*. While we presented some candidate genes at homologous 4A-QTL, further experimental analysis is needed. In addition to the coding features, the non-coding regions of the homologous regions were also found to support the evolutionary relationship that the comparative analysis presented. The results of this study have already shed light on our future cloning, editing, and engineering of some of the genes, proteins, and metabolites in wheat, barley, and oat.

## 5. Materials and Methods

### 5.1. Molecular Markers of OWBM and WSS Resistance Loci

Molecular markers delineating the major QTL conferring stem solidness against *WSS* oviposition (*Qss.msub-3BL* in hexaploid bread and *SSt1* in tetraploid wheat; 3BL-QTL hereafter) were taken from Nilsen et al. [12]. Markers linked to the *Sm1* locus, associated with resistance against *OWBM*, were taken from Kassa et al. [19]. Markers for two additional QTLs on 1A and 4A chromosomes in wheat contributing to *OWBM* resistance were taken from Hao et al. (for *QSm.hebau-4A*) and from Thambugala et al. (for *QSm.mrc-1A*), respectively [18,30]. Sequences of the molecular markers were retrieved from the wheat 90K array [46], CerealDB database (www.cerealsdb.uk.net, accessed on 23 September 2021), and Wheat URGI website (http://wheat-urgi.versailles.inra.fr/, accessed on 23 September 2021). For single nucleotide polymorphism (SNP) probes, both variants were kept.

### 5.2. Datasets Used in the Study

Cereal species with published genome sequences were used in this study. *T. aestivum* cv. Chinese Spring (hexaploid, AABBDD) IWGSC RefSeq v2.1 genome assembly and annotations were downloaded from Wheat URGI website (http://wheat-urgi.versailles.inra.fr/Seq-Repository, accessed on 21 July 2021; also available at NCBI project no. PRJNA669381). *T. turgidum* ssp. *durum* cv. Svevo (tetraploid, AABB) chromosome sequences, coding sequences and GFF files were downloaded from NCBI (BioProject: PRJEB22687, Assembly: GCA_900231444.1; https://www.ncbi.nlm.nih.gov/assembly/GCA_900231445.1 (accessed on 21 July 2021); LT934111.1[chr1A], LT934114.1[chr2B], LT934116.1[chr3B], LT934117.1[chr4A]). *T. turgidum* ssp. *dicoccoides* genotype Zavitan (tetraploid, AABB) chromosome sequences, coding sequences and GFF files were downloaded from NCBI (BioProject: PRJNA310175, Assembly: GCF_002162165.1; https://www.ncbi.nlm.nih.gov/assembly/GCF_002162155.1 (accessed on 21 July 2021); NC_041380.1[chr1A], NC_041383.1[chr2B], NC_041385.1[chr3B], NC_041386.1[chr4A]). *Hordeum vulgare* cv. Morex IBSC v2 chromosome, coding sequences, and GFF files were downloaded from EnsemblPlants (http://ftp.ebi.ac.uk/ensemblgenomes/pub/release-51/plants/fasta/hordeum_vulgare/, accessed on 14 November 2021). Similarly, *Oryza sativa* ssp. *japonica* cv. Nipponbare IRGSP 1.0 genome, coding sequences, and GFF files were downloaded from EnsemblPlants (http://ftp.ebi.ac.uk/ensemblgenomes/pub/release-51/plants/fasta/oryza_sativa/, accessed on 14 November 2021) [47]. *Secale cereale* line Lo7 pseudomolecules were downloaded from e!DAL IPK (https://doi.org/10.5447/ipk/2020/33 and https://doi.org/10.5447/ipk/2020/29, accessed on 14 November 2021) [35]. Lastly, *Avena sativa* v1.0 genome assembly was obtained from The Oat Genome Project website [29].

### 5.3. Homology Searches Using BLAST+

BLAST 2.11.0+ standalone package was used for all blast searches [48]. Blast databases were generated from chromosome or whole-genome sequences using makeblastdb. Marker sequences were blasted against these databases using blastn (-outfmt “6 std qlen slen”) and matches with at least 90% coverage of the marker sequences and at least 95% (for wheat genotypes), 90% (for barley and rye), 85% (for oat), or 80% (for rice) sequence identity to the genomic sequences were retained, based on evolutionary relationships. Markers that map to different locations with the same coverage and sequence identity were not used.

In cases where the number of molecular markers matching the genomic sequences above the filtering criteria given above did not allow identification of homologous regions, homologous transcripts were used instead. Specifically, for the wheat 3BL-QTL, transcript isoforms extracted from the homologous regions (detailed below) of *T. aestivum* Chinese Spring 3B (309 isoforms), *T. durum* Svevo 3B (316 isoforms), *T. dicoccoides* Zavitan 3B (258 isoforms), rye 6R (252 isoforms), and barley 3H (248 isoforms) chromosomes were combined (1383 isoforms in total). Rice IRGSP 1.0 CDSs were blasted against a database constructed from these combined transcripts (blastn; -evalue 1E-10, -outfmt “6 std qlen slen”). Matches were filtered for 80% sequence identity and 50% rice CDS coverage. Of the significant matches, only those that form a continuous interval on a chromosome were detained (this also compensates for the relatively low coverage threshold). Similarly, for wheat 2B-QTL, transcript isoforms from Chinese Spring 2B (452 isoforms), Svevo 2B (380 isoforms), Zavitan 2B (331 isoforms), and rye 7R (460 isoforms) were combined (1623 isoforms in total) and compared to all rice IRGSP 1.0 CDS and barley IBSC v2 CDS separately to define homologous regions in rice and barley, where molecular markers could not. For rice, filtering of the matches was conducted as above (80% identity, 50% coverage). For barley, the matches were filtered for 90% sequence identity and 80% barley CDS coverage, due to widespread conservation between wheat and barley genomes. Finally, for wheat 1A-QTL, Chinese Spring 1A (582 isoforms), Svevo 1A (545 isoforms), Zavitan 1A (559 isoforms), rye 1R (602 isoforms), and barley 6H (633 isoforms) chromosomes were combined (2921 isoforms in total). Rice IRGSP 1.0 CDSs were blasted against a database constructed from these isoforms and filtered as above.

For the wheat 4A-QTL, due to the scarcity of molecular markers saturating the putative QTL, 58 genes, from the earlier IWGSC v1.0 assembly, identified to reside within the resistance loci were taken in a recent publication [25]. The longest transcript isoforms from these genes were blasted against all coding sequences from Chinese Spring, Svevo, Zavitan, barley, rye, and rice (blastn; -evalue 1E-10, -outfmt “6 std qlen slen”). Matches with at least 80% coverage of the query transcript sequences and at least 95% (for wheat genotypes) and 90% (for barley and rye) or 80% (for rice, coverage threshold was also lowered to 50% due to increased evolutionary distance) sequence identity to the genomic sequences were retained.

For all resistance loci (3BL-QTL, 2B-QTL, 1A-QTL, and 4A-QTL), all transcripts identified in homologous regions in wheat, barley, rye, and rice were combined and compared against oat genome assembly (blastn; -evalue 1E-10, -outfmt “6 std qlen slen”). Due to the presence of introns in the genomic contigs, matches were considered significant if the percent sequence identity was above 85% over at least 300 aligned nucleotides.

To gain insight into probable functions of the genes within identified genomic loci, protein sequences encoded by these genes were compared against (1) all annotated and reviewed *Viridiplantae* proteins from the UniProt database (https://www.uniprot.org/; 40,927 sequences, accessed on 18 October 2021), (2) the annotated proteome of the model grass *Brachypodium distachyon* (v3.0; retrieved from EnsemblPlants https://plants.ensembl.org/Brachypodium_distachyon/Info/Index, accessed on 18 October 2021 [47]) and (3) iTRAQ-based proteomic data (unpublished) obtained from semi-solid stemmed wheat variety Scholar (PI607557) and solid stemmed wheat variety Conan (PI6907549) which both carry alleles associated with stem-solidness at *QSS.msub-3BL*. The matches were filtered for at least 50% sequence similarity and 50% coverage of the wheat, barley, rye, or rice protein for Uniprot blasts and at least 75% sequence similarity and 50% coverage for the *Brachypodium* proteome for closer evolutionary distance. Values for blast parameters, including similarity and coverage, are given in Supplementary Tables S2, S3, and S8, where, in specific cases, the significance of a match can be evaluated if needed. For the miRNA targets, since the above approach did not provide useful matches to either Uniprot or *Brachypodium* proteins in general, a further step was taken to compare these against all *Viridiplantae* proteins in the NCBI database web interface. The top 5 matches were provided to gain insight into the functions of these targets (Supplementary Table S7).

### 5.4. Extracting Transcripts from Homologous Regions

The borders for regions homologous to the *WSS* and *OWBM* resistance QTLs were determined using (1) molecular markers or (2) homologous transcripts, as detailed above. The transcripts within each region were then extracted using custom python3 scripts, using GFF files for the respective chromosomes, fasta files containing the coding sequences, and homologous region borders as input. Using the borders, all ‘gene’ features within the GFF file are listed, and the CDS IDs for each gene are outputted. In cases where a gene encodes several isoforms, only the longest CDS is retained. Isoforms were manually eliminated in cases where a homologous region was defined using homologous transcripts from the other species, as detailed above.

### 5.5. Comparative Analysis of Transcripts in Homologous Regions

For a comparative analysis of the organization of transcripts within the homologous regions, transcript sequences extracted from these regions as above were individually blasted against transcripts from the homologous region in Svevo for *WSS*-resistance loci (on 3B) and against transcripts from the homologous region in Chinese for *OWBM*-resistance loci (on 2B and 4A). Homologous transcripts were identified using blastn (-evalue 1E-10, -outfmt “6 std qlen slen”) and significant matches were filtered for at least 80% coverage of the marker sequences and at least 95% (for wheat genotypes), 90% (for barley and rye) or 80% (for rice) sequence identity. Visualization was performed with custom python3 scripts using matplotlib library.

### 5.6. Identification of Putative microRNAs and Target Sequences

Chromosomal sequences of the identified homologous regions were extracted from each respective chromosome with custom python3 scripts. *Viridiplantae* mature miRNA sequences were retrieved from miRBase (v21, June 2014) [49] to be used as a reference set. A fully automated miRNA pipeline, mirMachine, was used to carry out a homology-based in silico approach, as described in Cagirici et al., 2021 [50]. The pipeline uses National Center for Biotechnology Information (NCBI) Basic Local Alignment Search Tool (BLAST) to select for the candidate miRNA sequences at chromosome subsequences with at most 1 mismatch to the reference set. In the second step, mirMachine pipeline identifies the potential miRNA precursor sequences (premiRNAs) and fold them using RNAfold [51]. Potential premiRNA sequences are subjected to the following criteria: (1) no mismatches at Dicer cut sites, (2) no multi-branched loop at the hairpin with the mature miRNA sequence, (3) mature miRNA sequence could not be located at the head portion of the hairpin, (4) no more than four and six mismatches in miRNA and miRNA*, respectively [50,52,53,54].

Potential target transcripts of identified miRNAs from the homologous regions from each species were identified using mature miRNA sequences of each cereal crop in the psRNAtarget tool [55] against the high confidence coding sequences extracted from those loci. User customized schema option is used with expectation value set to be maximum 3 and a maximum UPE of 25. Protein sequences of the transcript targets mapped on the homologous regions are then protein-blasted against non-redundant protein sequences of *Viridiplantae* taxa using blast tool on (https://blast.ncbi.nlm.nih.gov/Blast.cgi, accessed on 24 August 2021).

## Data Availability

All data generated in this study are provided as Supplementary Materials. Customs scripts are available at request.

## Supplementary Materials

The following are available online at https://www.mdpi.com/article/10.3390/ijms222212349/s1.
